# Implication of Hippocampal Neurogenesis in Autism Spectrum Disorder: Pathogenesis and Therapeutic Implications

**DOI:** 10.2174/1570159X21666221220155455

**Published:** 2023-09-01

**Authors:** Chuanqi Liu, Jiayin Liu, Hong Gong, Tianyao Liu, Xin Li, Xiaotang Fan

**Affiliations:** 1 Department of Military Cognitive Psychology, School of Psychology, Third Military Medical University (Army Medical University), Chongqing, China;; 2 Battalion 5 of Cadet Brigade, Third Military Medical University (Army Medical University), Chongqing, China;; 3 Army 953 Hospital, Shigatse Branch of Xinqiao Hospital, Third Military Medical University (Army Medical University), Shigatse, China

**Keywords:** Autism, hippocampus, dentate gyrus, neurogenesis, stem cell, neurodevelopment

## Abstract

Autism spectrum disorder (ASD) is a cluster of heterogeneous neurodevelopmental conditions with atypical social communication and repetitive sensory-motor behaviors. The formation of new neurons from neural precursors in the hippocampus has been unequivocally demonstrated in the dentate gyrus of rodents and non-human primates. Accumulating evidence sheds light on how the deficits in the hippocampal neurogenesis may underlie some of the abnormal behavioral phenotypes in ASD. In this review, we describe the current evidence concerning pre-clinical and clinical studies supporting the significant role of hippocampal neurogenesis in ASD pathogenesis, discuss the possibility of improving hippocampal neurogenesis as a new strategy for treating ASD, and highlight the prospect of emerging pro‐neurogenic therapies for ASD.

## 1 INTRODUCTION

Autism spectrum disorder (ASD) is a cluster of heterogeneous neurodevelopmental disorders characterized by core features in two aspects: social communication deficits and repetitive sensory-motor behaviors [1]. The past years have witnessed a dramatic increase in the prevalence of ASD; nowadays, 1 in every 59 children has been diagnosed with ASD in the United States [2], with males being four times more likely to be diagnosed than females [3]. Due to the provision of support to patients who cannot function independently, which leads to higher costs in healthcare and school education, ASD has become a substantial social and economic burden. Behavior interventions and pharmacological treatments have been applied to ASD patients, yet they are currently limited to the treatment of co-occurring behaviors and not for ASD core symptoms. The benefits of therapeutic strategies for individuals with ASD are still limited. Therefore, there is an urgent need to develop new therapeutic options.

Both genetic and environmental factors could contribute to the occurrence of ASD [4]. Pre-clinical research works using different animal ASD models and clinical neuroimaging have also indicated several brain regions involved in the pathology of ASD, including but not limited to the hippocampus, amygdala, fusiform gyrus, inferior frontal gyrus, superior temporal sulcus, cerebellum, sensory cortices, and prefrontal region [5, 6]. Impairments in neurogenesis, excitatory/inhibitory balance, neurotransmitters, immune response, and functional connection in the above brain regions have been suggested to contribute to the ASD phenotype. Of note, the hippocampus, a region responsible mainly for memory and cognition, spatial reasoning, and pattern separation, has drawn increasing attention in ASD research [7-9]. Studies have indicated the hippocampus’s involvement in social behavior, a core aspect of the ASD phenotype [10, 11]. Abnormalities of hippocampal structure and function have been shown in several studies to contribute to the development of ASD [12]. Moreover, the hippocampus reaches major developmental milestones around two years of age, just as ASD symptoms typically become increasingly apparent [13]. Therefore, the Autism and Developmental Disabilities Monitoring network recommends that children with ASD be assessed at 36 months and start to receive community support and services by 48 months [14].

One of the typical features of hippocampal development is neurogenesis, a pool of neural progenitor cells (NPCs) enriched in the subgranular zone (SGZ) of the dentate gyrus (DG) that keeps generating new neurons. This process could last throughout the life span [15]. Emerging evidence has proved that deficits in hippocampal neurogenesis are involved in cognitive dysfunction and mood disorder and are closely related to several psychiatric disorders, such as depression, anxiety disorders, and ASD [16]. Accumulating evidence has indicated that promoting hippocampal neurogenesis could improve neurological conditions, such as depression, amyotrophic lateral sclerosis, Alzheimer’s disease, spinal cord injury, or Parkinson’s disease [17, 18]. It seems to be a promising option to remodel impaired and immature neurons forming pathological brain structures in ASD. Simultaneously, there is increasing evidence of the pro-neurogenic treatment of ASD [19, 20].

In this review, we will focus on current advances in the involvement of hippocampal neurogenesis in the pathogenic mechanism of ASD, discuss the possibility of improving hippocampal neurogenesis as a new strategy for treating ASD, and highlight the prospect of aiming at regulating DG neurogenesis as a potential therapeutic strategy.

## 2 HIPPOCAMPAL NEUROGENESIS

The hippocampus is a bilateral brain structure and plays a critical role in episodic memory, spatial reasoning, and social interaction [11, 21]. Postnatal and adult hippocampal neurogenesis have been unequivocally demonstrated in the DG of rodents and non-human primates, providing a particular type of structural plasticity to the brain [22, 23]. The SGZ is regarded as a secondary germinal zone expanding rapidly during the early 3^rd^ trimester of human gestational development [24-26]. After birth, the granule cell layer (GCL) of the DG of the newborn infant continues to grow and expand during the 1^st^ three months of postnatal life, and it peaks in size during the first week of postnatal development in mice. Neurogenesis during the fetal and early postnatal periods forms the DG and sustains activity-dependent continuous neurogenesis [27]. This ongoing generation of new neurons declines in adulthood and drops in old age [28].

**Fig. (1):**
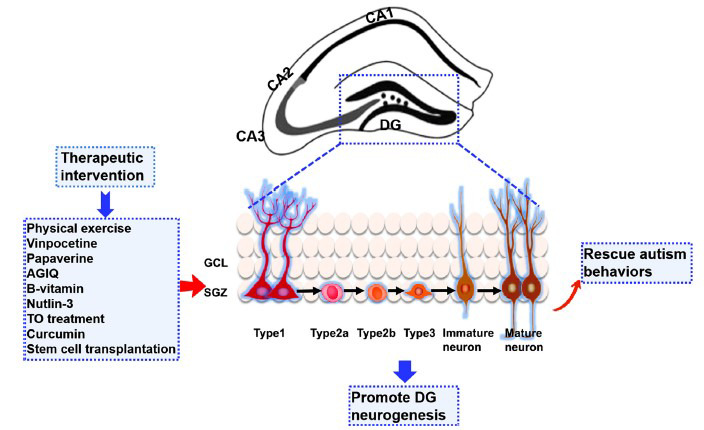
Multi-step processes involved in the DG neurogenesis and emerging pro‐neurogenic therapies for ASD.

A multi-step process schematizes the neurogenic niche (Fig. **1**). The radial-glia (RG) like NPCs (also known as type 1 cells) are located in the SGZ expressing specific markers, such as glial fibrillary acidic protein (GFAP), Nestin, SRY-box transcription factor 2 (SOX2), and brain lipid binding protein (BLBP) [29]. Type 1 cells undergo self-renewal and generate intermediate progenitors (Type 2 cells), consisting of non-fate-determined (Type 2a) and fate-determined (Type 2b). The intermediate progenitors then produce neuroblasts (Type 3), which are neuronally restricted and characterized by the expression of doublecortin (DCX), NeuroD1, and polysialic acid-neural cell adhesion molecule (PSA-NCAM), but capable of mitosis [30, 31]. Only a fraction of the neuroblasts lose their ability to divide; they survive and differentiate into dentate granule neurons that express phenotypic markers of mature neurons (neuronal nuclear antigen (NeuN), Calbindin, and Prox1) and send their dendrites into the molecular layer and their axons through the hilus toward the CA3 region to be integrated into existing neuronal circuits [32]. It takes approximately 30 days for a mature neuron to be generated from a Type 2a NPC. After producing multiple generations of neurons, the Type 1 and Type 2a progenitors start to produce astrocytes.

It has been indicated that these produced new neurons play critical roles in hippocampal physiology and neuronal plasticity [33]. Importantly, the functional integration of newborn neurons generated in the postnatal and adult hippocampus links to essential functions in spatial processing and pattern separation, as well as cognitive flexibility and reversal learning [34]. Of note, extrinsic and intrinsic factors influence the highly dynamic process of adult neurogenesis. It has been confirmed that factors, such as brain-derived neurotrophic factor (BDNF) and DCX, play a critical role in neurogenesis, synapse formation, neuroplasticity, as well as neuronal survival, thus influencing repetitive behavior and stereotypy, as indicated by a clinical study on Korean males and animal study in the BTBR T+Itpr3tfJ (BTBR) mouse model of ASD [35, 36]. BDNF also plays a fundamental role in synaptic transmission and plasticity in the hippocampus, representing a key regulator for long-term potentiation (LTP), learning, and memory. Dysregulation of BDNF expression and signaling, inducing changes in neuronal maturation and plasticity, is a hallmark of several neurodevelopmental diseases, such as attention-deficit hyperactivity disorder (ADHD), Rett syndrome (RTT), and ASD, suggesting that neuronal malfunction present in these disorders is suspected to be related to excessive or reduced BDNF levels [37].

Several key signaling pathways, such as Notch, Sonic hedgehog (Shh), and Wnts, growth and neurotrophic factors, cytokines, transcription factors, as well as epigenetic modifications, have been indicated to exert a modulatory effect on the distinct steps of adult hippocampal neurogenesis [38-42]. In the postnatal and adult brains, the Notch signaling pathway, as a fundamental signaling system, together with the Wnt/β-catenin, bone morphogenetic proteins, and Shh molecular signaling pathways contribute to the hippocampal stem cell regulation and they also govern the plasticity of the neural stem cells (NSCs) or NPCs, which are dedicated to the neurogenesis process [38]. They also modulate cell fate determination, axon growth, dendrite pruning and retraction, innervation patterning, and the expression of proteins vital for normal neuronal function, including neurotransmitters and ion channels. In the mature nervous system, these proteins dynamically control synaptic function and plasticity, while continuing to regulate neuronal cell survival [43]. The development of the newly produced neuron is based on the fine-tuning temporal activity level of transcription factors, which requires the coordinated synthesis of the diverse stage-specific proteins [44]. Moreover, results showed that epigenetic changes in the GABAergic system are essential for adult hippocampal neurogenesis in prenatally stressed mice [45].

## 3 ALTERED HIPPOCAMPAL NEUROGENESIS IN ASD PATIENTS

The global volume of the hippocampus increases rapidly until two years of age and continues to grow slowly after that. Symptoms of ASD typically emerge from 12 to 24 months of age, a time window related to critical developmental events within the hippocampus. Of note, the period between 18-24 months of age is considered a developmental milestone of the hippocampus. During this stage, newborn neurons in the DG and CA3 matured sufficiently to connect with the cerebral cortex and acquire adult-like typical morphology [46]. Given this overlap and addressed altered structure and function in the hippocampus of ASD individuals, it is suspected that hippocampal formation alterations may contribute to the development of ASD symptoms.

Existing studies on older children and young adults with ASD have demonstrated abnormalities in the hippocampus [47]. For volumetric alteration in the hippocampus, the enlarged hippocampal volume has been reported in children and adolescents with ASD [48]. The enlarged hippocampal structures in ASD patients may be due to pathological development or experience-dependent structural and functional plasticity [49]. However, several studies yielded contradictory and confusing results; reduced hippocampus volume or no significant differences compared to typically developing (TD) children have also been found [50]. The hippocampal volume appears to present multiple atypical changes within ASD, which might be explained by different measurement approaches and sample characteristics across studies, such as age and cognitive impairment. Another key reason for this variability may be possibly due to the heterogeneity of ASD, compromising different etiologies. Moreover, the right-left asymmetry in hippocampal volume development has been compared in TD and ASD children. Schumann *et al*. [51] have found that children with autism show a larger right hippocampal volume than TD controls. However, another study has reported that the left hippocampus was enlarged in ASD individuals compared to that in TD children [52]. Growing evidence indicates functional connectivity alterations related to the hippocampus within the first year of life in high-risk (HR) infants. One study using resting-state fMRI (rsfMRI) found that functional connectivity of networks underlying neural processing of language is disrupted in HR infants, characterized by lower intrahemispheric connectivity between the auditory cortex and higher-order temporal regions as well as the hippocampus [53]. Different from HR infants, low-risk (LR) infants displayed early specialization for human voice processing in the right temporal and medial frontal regions. Meanwhile, LR infants manifested higher sensitivity than HR infants to sad vocalizations in the right fusiform gyrus and left hippocampus [54]. Similar to a previous report determining globally weak functional connectivity in ASD youths, typically lower connectivity in the left hippocampal networks was also confirmed in ASD youths [55]. Rudie *et al.* [56] showed that compared with the TD children, ASD children had reduced functional connectivity in the neural circuits involving the left hippocampus and the right parahippocampal gyrus. This suggests that impaired memory function in ASD patients may be a possible reason for causing social deficits. It is suspected that unusual lateralization development of the hippocampus might be related to ASD individuals’ disabilities in social interaction and communication and restricted and repetitive behavior.

The neuropathology of the hippocampus in ASD subjects was first reported in a postmortem study in 1980. Abnormally small and compactly packed cells were observed in the subiculum and CA1 of the hippocampus [57]. Histologic abnormalities in DG have been well documented. Focal thickening of hippocampal CA1 and irregularities in the appearance of the DG were identified in three adult men with Fragile X syndrome (FXS). The anomalous growth of the dentate nucleus and dysplasia of the GCL in the DG, found in this report, imply abnormal neurogenesis in the hippocampus [58]. Compared with healthy controls, MRI demonstrated that a marked anatomical abnormality was detected in autistic patients in the area of dentata (DG+CA4) [59]. Groen *et al.* [60] noticed that the abnormal enlargement of the hippocampus was presented in adolescents with autism ages 12-18 years compared to healthy adolescents [60]. Moreover, one autopsy study identified abnormal DG neurogenesis, neuronal migration, and maturation in the ASD brain, which may account for the heterogeneity of the patient’s symptoms [61]. Subtle brain dysplasia of the DG with a scattered distribution of granule cells within the molecular layer and distorted GCL has been detected in a 13-year-old autistic boy. Post-mortem analysis of pathologic neuroanatomical changes in ASD patients points to impairments in neurogenesis. Additional genetic studies conducted on human tissue have emphasized the critical roles of hippocampal neurogenesis in contributing to the development of ASD.

Moreover, it has been further confirmed that those factors present in the blood of autistic patients markedly affected pre-programmed neurogenesis, neuronal proliferation, migration, differentiation, and circuit organization. Mazur-Kolecka *et al*. [62] found that sera from autistic children markedly decreased the proliferation of NPCs but promoted neuronal migration, development of small neurons with processes, and synaptic formation. It has been further confirmed that autoantibodies against NPCs appeared in sera of autistic subjects. Meanwhile, the autoantibodies in the serum against differentiating NPCs from autistic children might alter neuronal maturation [62]. Remarkably, mild oxidative stress inhibited differentiating NPC proliferation when treated with sera from autistic children, suggesting that altered NPC response to increased oxidative stress may contribute to the development of autism [63].

## 4 UTILIZING MOUSE MODELS TO UNDERSTAND THE IMPACT OF ASD ON HIPPOCAMPAL NEUROGENESIS

Animal models are essential for understanding the altered hippocampal neurogenesis responsible for ASD. Based on different generation methods, in the ASD field, animal models of ASD mainly include environment-induced models, idiopathic models, and genetic models that have been generated based on human mutations and phenotypes in an attempt to recapitulate the disease.

### 4.1 Reduced Hippocampal Neurogenesis in the Idiopathic ASD Model of BTBR Mice

The BTBR mouse strain, originally bred for studies on insulin resistance, diabetes-induced nephropathy, and phenylketonuria, is now one of the most widely used idiopathic models of ASD [64]. These animals display distinct behavioral patterns that resemble the core deficits of human ASD, including impaired social behavior and communication, increased repetitive behavior, and cognitive rigidity. The BTBR mouse hippocampus showed a marked reduction in the thickness of the GCL of the DG and thinning of the hilus [65]. Histopathological studies of the BTBR strain have confirmed that reduced hippocampal neurogenesis is evidenced by the significant reductions in the number of DCX, PSA-NCAM, and NeuroD immunoreactive cells in the SGZ, as well as a marked decline in the number of BrdU positive progenitors [65]. Additionally, a robust decrease in neuronal differentiation was confirmed in BTBR mice, evidenced by a reduced percentage of BrdU-positive cells colocalized with NeuN and an increased percentage of BrdU-positive cells colocalized with GFAP [65]. Given the critical role of BDNF in promoting the survival, differentiation, and proliferation of NPCs, BDNF mRNA level has also been confirmed in the hippocampus of BTBR mice using *in situ* hybridization [65].

### 4.2 Abnormal Hippocampal Neurogenesis in Environment-induced ASD Mouse Models

Valproic acid (VPA), a well-known antiepileptic/anticonvulsant drug, is commonly prescribed during human pregnancy. Maternal exposure to VPA has long been considered a risk factor for ASD susceptibility in offspring from epidemiological studies in humans [66, 67]. Several studies have found that prenatal and neonatal VPA exposure to various mammalian species, such as mice, rats, common marmosets, and ferrets, can result in autistic behaviors, including stereotypic and repetitive behavior, decreased sociability, reduced nociceptive reactivity, and impaired communication [68-71]. Though not all children of mothers exposed to VPA had a diagnosis of ASD, the timing of VPA exposure during gestation impacts the effects of VPA on behaviors [72, 73]. A maternal challenge with VPA in experimental animals has nonetheless been used extensively as a model of ASD to increase our understanding of the neurobiology underlying autistic behaviors. Importantly, prenatal and neonatal VPA exposure can alter hippocampus development, such as DG neurogenesis [70]. It is confirmed that utero VPA-exposed male Albino Wistar rats displayed lowered cAMP activity as well as pCREB level, and thus may define the decreased levels of DCX and BDNF, an indication of reduced hippocampal neurogenesis [74]. Following VPA exposure during the critical developmental window of the hippocampus, an inconsistency in the GCL neuron number of adult DG between rat and mouse studies has been described. Watanabe *et al.* [68] have found that developmental exposure to VPA mainly targets interneurons, followed by late influences on NPC proliferation in the SGZ and consequently increased GCL neurons in rat hippocampus. However, in mice, there is a decrease in newly formed neurons during the adult stage. Yochum *et al.* [75] reported that at 12 and 24 hours after VPA exposure to P14, the number of granule cells in the DG of the hippocampus of BALB/c mice was decreased due to VPA-induced apoptosis. Similarly, using mice prenatally exposed to VPA, Juliandi *et al.* [76] found that postnatal cognitive dysfunction is possibly related to the untimely augmentation of embryonic neurogenesis, which induced exhaustion of the NPC pool and subsequent inhibition of adult hippocampal neurogenesis. A ferret is a small laboratory animal that has morphological characteristics of the brain similar to humans and is a good model candidate because of its social and playful traits. One recent study has indicated that VPA exposure to ferret infants induced disruption in social behavior. Meanwhile, it promoted the proliferation of neuron progenitors in the DG, and consequently brought extra NPCs into the DG subgranular layer [70].

Inflammation during pregnancy and perinatal infections are well-identified environmental risk factors, leading to ASD. Increased cytokines and activated microglia were found in the postmortem brain of ASD subjects. It is well-known that lipopolysaccharide (LPS), a cell wall component of gram-negative bacteria, leads to late-gestation maternal immune activation (MIA) that results in ASD-like behaviors in the offspring [77]. Similarly, neonatal LPS-treated rats at P3 induced acute pro-inflammatory responses in the brain, resulting in ASD-like behaviors and impaired hippocampal neurogenesis, serving as an ASD model [78]. Fractalkine signaling (CX3CL1/CX3CR1), a major neuron-to-microglia communication pathway, is involved in the pathogenesis of ASD induced by the immune challenge. An association of the variant of CX3CR1 with the ASD phenotype has been reported [79]. Meanwhile, CX3CR1 KO mice could model ASD-like behaviors, such as deficits in social interaction and increased repetitive behavior phenotypes [80]. Importantly, CX3CR1 KO mice displayed a reduction in neurogenesis in the DG *via* phagocytosis [81]. Loss of CX3CR1 in microglia could disrupt DG neurogenesis, reduce the number of dendritic spines as well as impair the synaptic integration of adult-born neurons [82]. This might explain a reduced CX3CR1 expression in MIA offspring involved in ASD-like behaviors with alerted synaptic pruning [83]. One recent study has reported that severe inflammatory pain in neonates is related to the development of ASD in juveniles. Meanwhile, reduced DG neurogenesis occurred in P21 juvenile rats subjected to inflammatory pain and related to IL-1β increase in the brain. These novel observations of Lee *et al.* [84] suggested that severe inflammatory pain in neonates and persistent inflammatory responses may reduce hippocampal neurogenesis, delay neuronal maturation, and ultimately contribute to psychiatric disorders, such as ASD. The alleviation of pain stimulus and inhibition of inflammation activities during development seem extremely important in blocking the possible progression of ASD [84].

Furthermore, epidemiological and experimental evidence document that gestational exposure to air pollution increases the risk of ASD. Cole *et al.* have demonstrated that gestational exposure to urban-derived nanosized particulate matter decreased neurogenesis in the DG, evidenced by a marked decrease in the number of EdU^+^/NeuN^+^ cells [85]. Another study reported that gestational exposure to PM2.5 caused autism-like behaviors, such as social communication deficits and stereotyped repetitive behavior, and inhibited hippocampal neurogenesis [86]. Consistent with these reports, we found that mice exposed to silica nanoparticles (SiO2-NPs) at higher doses during the first postnatal week resulted in a significant decrease of NPC proliferation in the DG-SGZ and exhaustion of the RGC population labeled with GFAP^+^/SOX2^+^ [87]. We further confirmed that mice exposed to 20 mg of SiO_2_-NPs exhibited defective social behavior deficits and slight anxiety-like behaviors in adulthood [87]. Thus, these potential environmental hazards have neurotoxic effects on DG neurogenesis and subsequently contribute to the development of autism-like behaviors in adults.

Perinatal medication exposures, such as selective serotonin reuptake inhibitor medications (SSRIs), the first lines of treatment for maternal affective disorders, are related to ASD risk in children [88]. Several meta-analyses indicated a significant positive association between in-utero exposure to SSRIs and ASD. Yet this association does not necessarily reflect a causal relationship since the results included in these meta-analyses are likely affected by other confounding factors, such as underlying maternal psychiatric disorders [89-92]. Besides, Ames *et al.* [93] found that maternal psychiatric conditions but not taking SSRIs during pregnancy were related to increased danger of neurodevelopmental disorders in offspring. On the other hand, emerging evidence from animal studies suggested that prenatal SSRI exposure might negatively affect social behavior. Zahra *et al.* observed abnormal behaviors, such as anxiety, altered locomotion, and disordered social interactions in 2-5 months old offspring with prenatal citalopram exposure [94]. Fluoxetine exposure throughout gestation and early lactation resulted in deficits in sociability and social novelty-seeking behavior in the juvenile offspring, which persisted into young adulthood [95]. One study has reported that perinatal fluoxetine treatment avoided effects induced by maternal stress on sibling play behavior, increased socially aggressive behavior, and reduced time grooming with a novel con-specific in males [96]. Qiu *et al.* [97] have confirmed that postpartum maternal fluoxetine treatment led to reduced proliferative DCX-positive cells in males and DCX-labeled post-mitotic cells in both sexes in the ventral DG.

### 4.3 Abnormal Hippocampal Neurogenesis in Genetic ASD Mouse Models

Advancement in human genetics has led to the discovery of many genes responsible for ‘monogenic’ forms of ASD. Studying these genes in pre-clinical models helps understand critical neurobiological pathways involved in ASD pathogenesis. Thus, over 1000 autism genetic mouse models and over 200 rat genetic models have been created. Some high-ranking candidate risk genes for ASD, such as calcium-dependent activator protein for secretion 2 (CAPS2), Engrailed-2 (EN2), Phosphatase and tensin homolog (Pten), Liver X receptor β (LXRβ), Topoisomerase 3β (Top3β), Methyl cytosine binding protein 2 (MECP2), Methyl-CpG binding protein 1 (MBD1), and Tbx1, are involved in the modulation of DG neurogenesis (Table **1**).

#### 4.3.1 CAPS2

The human CAPS2 gene is located on chromosome 7q31.32, within the autism susceptibility locus, in single alleles of autistic patients [98]. Sadakata *et al.* [99] showed decreased transcription of CAPS2 in autistic brains; CAPS2 was found to be highly expressed in the hippocampal DG region, related to BDNF secretion. Caps2 KO mice not only have deficits in neuronal development and survival but also display deficits in social interaction, social communication, and hyperactivity, which are reminiscent of the behaviors of autistic patients [100, 101]. It is revealed that the Caps2 gene deficit influences adult hippocampal neurogenesis and the maturation of newborn neurons induced by the environmental enrichment condition [102]. BDNF plays a critical role in the postnatal development of forebrain GABAergic neurons, including the development of inhibitory neurons and their circuits and the altered GABA signaling, which has been supposed to be involved in the symptoms of ASD. BDNF secretion in the hippocampus of Caps2-KO mice secretion is reduced, and GABAergic system function is impaired, as evidenced by decreased late-phase LTP at CA3-CA1 synapses, reduced theta oscillation frequency in the hippocampus, and increased anxiety-like behavior [103].

#### 4.3.2 EN2

Genome-wide association studies revealed that EN2 is a homeobox transcription factor, functions as a patterning gene in early development, and is a candidate gene for ASD [104-106]. Mice lacking *En2* display ASD-like behavioral traits, such as decreased sociability, spatial learning deficits, and increased seizure susceptibility [107]. The *En2*-KO mice displayed reductions in hippocampal volume and cell numbers due to aberrant neurogenesis characterized by excess proliferation in the early Sox2^+^/Tbr2^+^ progenitors, increased apoptosis in differentiating neuroblasts, and reduced newborn neuron survival [108]. Notably, En2 functions in hippocampal NPCs by inhibiting proliferation and promoting survival and differentiation in a cell-autonomous manner [109].

#### 4.3.3 Pten

*PTEN*, located on chromosome 10 (10q23.3), is closely related to autism and accounts for 5-17% of cases of autism [110]. Mice with deleted *Pten* in the mature neurons of the cerebral cortex and hippocampus displayed deficits resembling certain features of human ASD [111]. Deleting *Pten* in adult NSCs leads to a higher proliferation rate and accelerated differentiation of the stem/progenitor cells, resulting in the depletion of the NSC pool and increased differentiation toward the astrocytic lineage at later stages [112]. Additionally, mice with conditional *Pten* deletion in adult NSCs have enlarged DG and exhibit impairments in social interactions and seizure activity resembling certain features of human ASD [112]. One recent study has further confirmed that cerebrovascular-specific deletion of *Pten* disrupts adult neurogenesis with accompanying lactate accumulation [113].

#### 4.3.4 Fmr1

FXS is a monogenic syndrome caused by transcriptional silencing of the *Fmr1* gene and a single-gene cause of autism, impairing the translation of Fragile X mental retardation protein (FMRP) [114]. The *Fmr1*-KO mouse has a behavioral phenotype that resembles ASD symptoms, such as social deficits, increased repetitive behavior, hyperactivity, and learning deficits [114-116]. Luo *et al*. [117] have confirmed specific mRNAs modulated by *Fmrp* in the proliferation and differentiation of stem cells, including glycogen synthase kinase 3β (GSK3β), which has been implicated in adult neurogenesis. This study reports a novel role for Fmrp in the regulation of adult neurogenesis and supplies a direct indication that altered adult neurogenesis could be involved in the pathogenesis of fragile X mental retardation [117]. Meanwhile, Guo *et al*. [118] found that deletion of *Fmrp* in adult NSCs by inducible gene recombination led to increased proliferation and altered fate specification of NSCs and significantly damaged hippocampus-dependent learning in mice. On the other hand, restoration of *Fmrp* expression typically in adult NSCs rescued learning dysfunctions in *Fmrp* KO mice [118]. Some studies have demonstrated that Fmrp plays a role in stem cells, including adult NSCs in the hippocampal DG. Eadie *et al*. [119] found that loss of *Fmr1* in mice led to anxiety-related behaviors and produced alterations in adult hippocampal neurogenesis [119]. Besides, adult *Fmr1* KO mice displayed an increased rate of progenitor cell proliferation, altered fate specification, and diminished medial perforant path-granule cell LTP [117, 118, 120].

#### 4.3.5 CYFIP1

Copy number variation (CNV) at the 15q11.2 region has been identified as a significant risk locus for autism, including a gene coding for CYFIP1 (cytoplasmic FMR1 interacting protein 1) [121]. *CYFIP1* haploinsufficiency in mice led to impairments in social behavior and motor learning and defects in dendritic spine morphogenesis [122]. One recent work found that *CYFIP1* haploinsufficiency increased adult-born hippocampal neurons due to a specific deficit in microglia-induced apoptosis. Meanwhile, these newly produced neurons displayed migration deficits due to abnormal Arp2/3 activity and actin cytoskeleton remodeling [123].

#### 4.3.6 LXRβ

LXRs, including LXRα and LXRβ, two isoforms, play critical roles in the nervous system, such as promoting NSC proliferation and neuronal differentiation, improving synaptic plasticity and function, preventing neurodegeneration, inhibiting inflammation as well as regulating cholesterol homeostasis in the brain [124-126]. Our previous studies have verified that LXRβ is essential for RGC development and laminated CNS structures [127-131]. We noticed that LXRβ KO mice displayed impaired DG development, including deficits in the formation of progenitor cells and granule cell differentiation [127]. Analysis of the proliferation and differentiation of cultured NPC revealed that T0901317 (TO), a potent LXR agonist, increased NPC proliferation and prompted NPC differentiation toward neurons [127, 131]. We also found that LXRβ deletion in mice led to autistic-like behaviors, including social interaction deficits, increased repetitive behavior, and impairment in reversal learning. These data revealed a central role for LXRβ in DG neurogenesis, explaining its association with the genesis of autism-related behaviors in LXRβ-deficient mice [131].

#### 4.3.7 Top3β

Top3β, an RNA topoisomerase, biochemically and genetically interacts with FMRP and is involved in ASD pathogenesis [132]. Top3β mutant mice displayed increased latency before the first exploration with a target mouse, which suggested a deficit in social interactions, a hallmark of autism [133]. These mice also exhibited impaired hippocampal neurogenesis and synaptic plasticity [133]. Top3β mutation in flies and mice caused deficits in synapse formation, with similar findings in FMRP mutant flies and mice [134]. Top3β-KO mice were found to have greater generalized anxiety levels, and the impaired synaptic plasticity phenotype resembled those observed in patients. Top3β deletion in mouse impaired hippocampal neurogenesis and synaptic plasticity [135, 136].

#### 4.3.8 MECP2

MECP2 is a structural chromosomal protein and is important for neuronal system development. Mutations in the gene encoding *MECP2* were previously associated primarily with RTT [137], but a recent study by Wen *et al.* has identified genetic mutations of the MECP2 gene in autism patients [138]. Newly produced granule cells in adult DG resembled maturational defects observed at early postnatal ages. As a result, they might not integrate appropriately into the existing circuit of the adult hippocampus [139]. Duplications of *MECP2*-encompassing segments result in the *MECP2* duplication syndrome, which is associated with severe autism [140]. Abnormal DG neurogenesis occurred in *MECP2* transgenic mice, as evidenced by adult hippocampal quiescent NPCs, and significantly accumulated in transgenic mice compared to wild-type (WT) mice. The reduced NPCs and increased neuroblasts were also observed in the adult hippocampus of *MECP2* transgenic mice [141, 142].

#### 4.3.9 MBD1

MBD1 mediates gene expression through methylated DNA binding and epigenetic regulation [143]. Abnormal epigenetic pathways have been identified to be involved in several neurodevelopmental disorders, including autism. Deletion of MBD1 in mice results in *core autism*-related *deficits and* relevant behaviors, such as deficits in social interaction and learning, anxiety-like behavior, impaired sensory-motor gating, depression-like behavior, and aberrant brain serotonin activity [143]. Furthermore, epigenetic regulation contributes to keeping the self-renewal and multipotency of adult stem cells. MBD1 is expressed in the NSC of the adult DG, suggesting its role in hippocampal neurogenesis [144]. Deletion of MBD1 in mice was found to promote the accumulation of undifferentiated NSC and defective neuronal lineage differentiation. Transcriptome analysis of NSCs/NPCs dissected from the mouse DG has identified that gene sets associated with astrocyte lineage genes were up-regulated in cells from MBD1 mutant mice [144, 145].

#### 4.3.10 Tbx1

Hemizygous deletion at human 22q11.2 is identified as related to ASD [146]. *Tbx1* encodes a transcription factor and is a determining gene among 30-40 contiguous genes in a common deletion of a 22q11.2 hemizygous segment [147]. *Tbx1* heterozygous mice display ASD-like behaviors, including deficits in reciprocal social interaction and communication, impaired working memory, increased repetitive behaviors, and heightened anxiety-related behavioral traits [148]. Tbx1 protein expression is found to be enriched in adult NPCs of the adolescent mouse brain [148]. Moreover, copy number elevations of catechol-O-methyl-transferase or *Tbx1* reduced the proliferation of adult NSCs/NPCs in a cell-autonomous manner *in vitro* and the migration of their progenies in the hippocampus GCL *in vivo [**149**].*

## 5 HIPPOCAMPAL NEUROGENESIS-BASED REGENERATIVE THERAPY PROPOSED FOR THE TREATMENT OF ASD

The exceptional role of hippocampal neurogenesis involved in cellular and behavioral plasticity is confirmed by the evidence that impaired neurogenesis is tightly coupled to autistic deficits in social memory, cognitive flexibility, and repetitive behavior [17, 150-152]. Similarly, restoration of hippocampal neurogenesis through lifestyle or therapeutic interventions can rescue autistic behaviors [153, 154]. Restoring hippocampal neurogenesis has emerged as a promising therapeutic intervention in ASD [155].

It is well-documented that physical exercise alleviates memory dysfunction, rescues social deficits, and relieves hyperactivity in autism [156]. In a separate study, Seo *et al*. [157] found that treadmill exercise for four weeks, starting postnatal day 28, reduced aggressive tendency and prompted the correct decision in spatial learning and memory in the VPA-injected rats. Meanwhile, treadmill exercise increased DG neurogenesis in the VPA-injected rats, which might contribute to the alleviation of autism-like symptoms. Javadi *et al*. [158] found that young adult *FMR1*-deficient mice treated with Nutlin-3 for five injections produced a long-lasting therapeutic effect on hippocampal neurogenesis and cognitive function *via* modulating the adult NSC niche.

Vinpocetine, a specific inhibitor of the phosphodiesterase-1 enzyme, is known to exert neuroprotective properties [159]. One recent study has reported that adult male rats prenatally exposed to VPA administrated with vinpocetine displayed amelioration of hyperlocomotion, social deficits, stereotypy, anxiety, and nociceptive changes [36]. Furthermore, the increased level of DCX in DG by vinpocetine administration seemed to be involved in these behavioral alterations [36]. Vinpocetine may be a consideration for future investigation in the clinical setting. The PDE10A inhibitor, papaverine, has been confirmed to rescue social deficits and stereotypy in miR-137^flox/+^; Nestin-Cre mice and repair neuronal morphological alterations [160]. In addition, the administration of papaverine to Pre-VPA animals from P21 to P48 resulted in improvements in social behavior and corrected repetitive behavior, anxiety, locomotor, and nociceptive changes [161]. Moreover, papaverine treatment resulted in a significant increase in the levels of DCX of the Pre-VPA group, which was associated with the alleviation of the core behaviors of ASD [74].

LPS-exposed neonatal animals revealed detrimental effects in hippocampal neurogenesis from puberty to adulthood and showed the progression of ASD-like behaviors [162]. Alpha-glycosyl isoquercitrin (AGIQ) exerts chemo-preventive effects, such as antioxidant and anti-inflammatory effects. It has been further confirmed that continuous AGIQ treatment, starting during late gestation, ameliorated progressive autistic behavior deficits and suppressed DG neurogenesis by critically inhibiting both inflammatory and oxidative responses caused by LPS [78]. B-vitamin is an indispensable nutrient to the human body and possesses anti-inflammatory and antioxidative properties [163]. Consistent with this study, B-vitamin supplementation to pregnant mice exposed to PM2.5 alleviated the autistic-like behaviors of offspring mice by enhancing anti-inflammatory and antioxidant capacities as well as improving hippocampal neurogenesis [86]. It was found that B-vitamin supplementation exhibits long-lasting effects on mitigating neurodevelopmental abnormalities induced by gestational exposure to PM2.5 [86]. These studies implied that embryonic treatment results in lasting improvements in autistic phenotypes, potentially through structural repair.

It is well documented that the first 2 weeks after birth in mice, equivalent to the period from the last trimester of pregnancy to the first few years after birth in humans, is the critical period for DG development [164]. It has been indicated that early postnatal treatment potentially repairs the structure, which is associated with continuous symptom alleviation [165]. Our study has demonstrated that postnatal treatment with TO, typically activated endogenous LXRβ and its target genes in the hippocampus and rescued the social defects and stereotypical behaviors in adult BTBR and VPA-induced autism mouse models. Importantly, we further confirmed that early postnatal TO treatment could enhance hippocampal neurogenesis [155]. Curcumin, a natural herbal component, has been confirmed to exert potent anti-inflammatory, antioxidant, and antineoplastic effects in neurological and psychological disorders [166]. Remarkably, curcumin has been demonstrated to exert neuroprotective properties *via* enhanced hippocampal neurogenesis. We demonstrated that neonatal curcumin treatment from P6-P8 significantly alleviated social deficits, repetitive behavior, and cognitive impairments in BTBR mice [17]. We further confirmed that neonatal curcumin treatment efficiently rescued hippocampal neurogenesis by preventing the exhaustion of the NPC pool and boosting NPC differentiation in BTBR mice [17].

Growing studies have indicated that gut dysbiosis might be involved in the increased vulnerability of ASD development [167, 168]. Liu *et al.* [168] revealed that antibiotic-induced gut microbial alteration in newborn mice resulted in ASD-like behavior impairment, which is related to a reduction in adult neurogenesis. Remarkably, the reconstruction of gut microbiota with healthy gut flora exerted therapeutic effects against adult neurogenesis and impaired behaviors in the dysbiosis mice [168]. This highlights the prospect of microbiome-mediated treatment for ASD individuals.

## 6 STEM CELL THERAPY IN THE TREATMENT OF ASD

A growing number of studies highlight that stem cell transplantation possesses a therapeutic potential for patients affected with incurable neurological disorders [169-171]. In the context of ASD, the evidence indicates that stem cell treatment markedly improves the ASD rating scale, abnormal behavior checklist scores, and clinical global impression evaluation, suggesting its potential benefit in treating children diagnosed with ASD [172, 173]. Up to now, no serious adverse events associated with stem cell therapy have been found, regardless of the cell source, dosage, and delivery routes [174-176]. It has been reported that 25 autistic children displayed behavioral improvements within 6 months of receiving umbilical cord blood-derived cell therapies, and adverse event assessments during the 12 months after injection showed that the treatment was safe and well tolerated [177]. Besides, Dawson *et al*. [178] found that autistic children without intellectual disability (ID), who received allogeneic cord blood infusion showed behavioral improvements, but a single infusion of cord blood was not associated with improved socialization skills or reduced autistic symptoms. It was also found that cord blood infusion was safe and well tolerated. Of note, this research is limited by a small sample size and restricted age and dosing ranges, and the safety of stem cell therapy certainly needs further investigation in larger cohorts.

Despite the gap between animal models and ASD patients, a growing number of preclinical studies showed that stem cell therapy could alleviate ASD symptoms by improving hippocampal neurogenesis.

Bone marrow-derived mesenchymal stem cells (MSCs) are multipotent stem cells typically differentiating into mesenchymal lineages, such as bone, cartilage, and fat. It is believed that MSC does have the potential to support and enhance endogenous neurogenesis and represent an attractive cell source for ASD treatment. *In vitro*, hippocampal neurogenesis has been dramatically increased in the presence of MSCs. Intracerebral transplantation of MSC can enhance hippocampal neurogenesis and improve hippocampal-related behaviors and function by induction of neurotrophic factors and immunomodulation [179]. Thus, MSC transplantation is speculated to be a potential therapy for ASD *via* enhanced hippocampal neurogenesis. Indeed, transplantation of MSCs into the cerebral lateral ventricles of BTBR mice resulted in the alleviation of ASD-like behaviors, such as a reduction of stereotypical behavior and impairments in cognitive flexibility and social behavior [180]. Meanwhile, enhanced hippocampal neurogenesis was identified in BTBR mice by MSC transplantation, as observed with increased Ki67-labeled NPCs and DCX-positive neurons in the DG [180]. It is well documented that the enhanced hippocampal neurogenesis by MSC transplantation is related to paracrine secretions, such as BDNF, IGF-1, NGF, and VEGF. As expected, an increased BDNF protein level in the hippocampus was also observed in MSC-transplanted mice [180]. Likewise, the intranasal delivery of human umbilical cord-MSC exosomes was found to efficiently alleviate the social deficit and repetitive, stereotyped behavior in the offspring of VPA-treated mice [179]. This is further supported by the finding that BTBR mouse transplantation with induced MSC that secreted high levels of neurotrophic factors had significant advantages over MSC transplantation in terms of improving communication skills and stereotypic behavior for as long as 6 months post-treatment [180]. Similarly, Gobshtis *et al*. [181] demonstrated that intracerebral transplantation of MSC efficiently rescued cognitive and social behavior deficits in VPA-exposed mice. Meanwhile, impaired hippocampal neurogenesis was also rescued by MSC transplantation. Furthermore, a statistically significant correlation between neuronal differentiation and behavioral scores suggested that increased hippocampal neurogenesis involved in improved ASD-like behaviors was due to MSC transplantation [182].

Noticeably, many investigations have found that human amniotic epithelial cells (hAECs) isolated from the layer closest to the fetus represent a unique class of stem cells [20]. Moreover, hAECs can exert neuroprotection in models of CNS disorders, such as stroke, perinatal hypoxic-ischemic brain injury, and fetal brain injury [183]. Our study has confirmed that intraventricular injection of hAECs into adult male BTBR mice could ameliorate social deficits significantly. In addition, hAEC transplantation restored the decline of neurogenesis, evidenced by increased BrdU-positive cells, DCX-positive neurons, Prox1-positive neurons, and GFAP^+^/Sox2^+^ double-stained NPCs in the hippocampus of BTBR mice. Increased levels of BDNF and TrkB in the hippocampus might be involved in the beneficial effects caused by hAEC transplantation [20].

A concerning issue is that the heterogeneity of ASD has implications for treatment efficiency. Current pro-neurogenic stem cell treatments were attempted in mouse models; however, whether they have the same impact on ASD patients remains to be investigated.

## CONCLUSION

Based on pre-existing studies, impaired hippocampal neurogenesis in established ASD models may underlie some of the abnormal behavioral phenotypes seen in ASD, but only little is known about its potential significance in this context. Interestingly, interventions to regulate hippocampal neurogenesis and thereby reconstruct the hippocampal network in animal models present an effective neuroprotective strategy, evidenced by some ASD-like behavioral traits partially or completely rescued. The clinical application of pro‐neurogenic strategies in humans has yet to be demonstrated. Innate hippocampal neurogenesis is finite in type and scale, and pathologic factors involved in ASD might markedly influence the outcome of pro‐neurogenic treatments. The cellular and molecular pathways typically related to the neurogenic niche regulation mediating the pro‐neurogenic therapeutic effects on ASD need to be understood clearly. In the future, it is necessary to conduct additional, large-scale pre-clinical and clinical studies to illustrate the safety and efficiency of therapies targeting hippocampal neurogenesis for ASD.

## Figures and Tables

**Table 1:** Altered hippocampal neurogenesis in mouse models of ASD.

**Mouse Model**	**Hippocampus Neurogenesis**	**References**
8 to 10-week-old BTBR mice	Neurogenesis↓, DCX^+^↓, PSA-NCAM^+^↓, NeuroD^+^↓, BrdU^+^↓, BrdU^+^/NeuN^+^↓, BrdU^+^/GFAP^+^↑, BDNF mRNA↓	[65]
VPA-exposed ferret infants	Neurogenesis↑, BrdU^+^↑, BrdU^+^/SOX2^+^↑	[70]
Utero VPA-exposed male albino Wistar rats	Neurogenesis↓, DCX↓, BDNF↓, pCREB↓, synapsin-IIa↓	[74]
Prenatal VPA-exposed mice	Embryonic neurogenesis↑, NPCs↓, adult neurogenesis↓, BrdU^+^↓, Ki67^+^↓, BrdU^+^/NeuN^+^↓, BrdU^+^/S100β^+^↓	[76]
Rats exposed to VPA at P13 and P14	Neurogenesis↓, DCX^+^↓, BrdU^+^↓	[155]
Rats exposed to LPS at P3	Neurogenesis↓, P21(NeuN^+^**↓**), P77(DCX^+^↓, TUBB3^+^↓, PCNA^+^↑)	[78]
*Cx3cr1* KO mice	Neurogenesis↓, DCX^+^↓, dendritic complexity**↓**	[81]
Perinatal SSRIs treated rats	Neurogenesis↓, males: proliferative DCX^+^↓; female: post-mitotic DCX^+^↓	[97]
Adult *Caps2* KO mice	Environmental enrichment-induced adult neurogenesis↓, BrdU^+^↓, BrdU^+^/calretinin^+^↓, BrdU^+^/NeuN^+^**↓**	[102]
*Caps2* KO mice	BDNF**↓**, GABAergic neurons**↓**, synapses↓	[103]
*En2*-KO mice	NPC proliferation↑, BrdU^+^/Sox2^+^**↑**, BrdU^+^/Tbr2^+^**↑**; cell death↑, caspase-3^+^/DCX^+^**↑**	[108]
*Nestin-creER^T2^; Pten^loxp/loxp^* mice	Neurogenesis**↑**, RGL pool↓, Ki67^+^**↑**, BrdU^+^/NeuN^+^**↓**, BrdU^+^/S100β^+^↑, BrdU^+^↑	[112]
*SP-A-Cre; PTEN^flox/flox^* mice	NPC proliferation↑, BrdU^+^**↑**, Sox2^+^/GFAP^-^/BrdU^+^**↑**, Sox2^+^/GFAP^+^/BrdU^+^↓; neurogenesis↓, DCX^+^↓ BrdU^+^/NeuN^+^↓	[113]
*Fmr1* KO mice	BrdU^+^**↑**, BrdU^+^/NeuN^+^↓, BrdU^+^/S100β^+^**↑**	[117]
*Fmr1^loxP/y^: Nes-CreER^T2^* mice	Neurogenesis↓, RGC**↑,** DCX^+^↓, NeuN+↓S100β^+^/GFAP^+^**↑**	[118]
*Cyfip1* heterozygous KO mice	Neurogenesis**↑**, DCX^+^**↑**, caspase3^+^↓, BrdU^+^/NeuN^+^**↑**	[123]
*LXRβ* KO mice	Neurogenesis↓, Sox2^+^/GFAP^+^↓, BLBP^+^/GFAP^+^↓, BrdU^+^↓, Sox2^+^↓, DCX^+^↓, Nestin^+^↓, Prox1^+^↓, Tbr2^+^↓	[131]
*Top3β* KO mice	BrdU^+^↓, DCX^+^↓	[133]
*MECP2* transgenic mice	Neurogenesis↓, BrdU^+^↓, GFAP^+^/Nestin^+^/Ki67^−^**↑** GFAP^-^/Nestin^+^/Ki67^+^↓, BrdU^+^/DCX^+^**↑**,	[142]
*MBD1* KO mice	aNSCs**↑**, BrdU^+^**↑**, Tuj1^+^↓, GFAP^+^↓, NeuroD1 mRNA↓, Tuj1 mRNA↓, GFAP mRNA↓	[144]
	Nestin^+^**↑,** Nestin^+^/DCX^+^**↑,** Nestin-/DCX^+^↓	[145]
Tbx1-EGFP transfected mice	Migration and proliferation of adult neural stem/progenitor cells↓	[149]

**Note**: (↑ = increased, ↓ = decreased).

## LIST OF ABBREVIATIONS

ADHD: Attention-deficit Hyperactivity Disorder
AGIQ: Alpha-glycosyl Isoquercitrin
ASD: Autism Spectrum Disorder
BDNF: Brain-derived Neurotrophic Factor
BLBP: Brain Lipid Binding Protein
DG: Dentate Gyrus
FXS: Fragile X Syndrome
GCL: Granule Cell Layer
GFAP: Glial Fibrillary Acidic Protein
hAECs: Human Amniotic Epithelial Cells
ID: Intellectual Disability
MSCs: Mesenchymal Stem Cells
NPCs: Neural Progenitor Cells
RTT: Rett Syndrome
SGZ: Subgranular Zone
TD: Typically Developing
